# Transfer Learning Based Semantic Segmentation for 3D Object Detection from Point Cloud

**DOI:** 10.3390/s21123964

**Published:** 2021-06-08

**Authors:** Muhammad Imad, Oualid Doukhi, Deok-Jin Lee

**Affiliations:** 1 Center for Artificial Intelligence & Autonomous Systems, Kunsan National University, 558 Daehak-ro, Naun 2(i)-dong, Gunsan 54150, Korea; imadsafi08@kunsan.ac.kr (M.I.); doukhioualid@kunsan.ac.kr (O.D.); 2 School of Mechanical Design Engineering, Smart e-Mobilty Lab, Center for Artificial Intelligence & Autonomous Systems, Jeonbuk National University, 567, Baekje-daero, Deokjin-gu, Jeonju-si 54896, Korea

**Keywords:** 3D object detection, point cloud processing, transfer learning, semantic segmentation

## Abstract

Three-dimensional object detection utilizing LiDAR point cloud data is an indispensable part of autonomous driving perception systems. Point cloud-based 3D object detection has been a better replacement for higher accuracy than cameras during nighttime. However, most LiDAR-based 3D object methods work in a supervised manner, which means their state-of-the-art performance relies heavily on a large-scale and well-labeled dataset, while these annotated datasets could be expensive to obtain and only accessible in the limited scenario. Transfer learning is a promising approach to reduce the large-scale training datasets requirement, but existing transfer learning object detectors are primarily for 2D object detection rather than 3D. In this work, we utilize the 3D point cloud data more effectively by representing the birds-eye-view (BEV) scene and propose a transfer learning based point cloud semantic segmentation for 3D object detection. The proposed model minimizes the need for large-scale training datasets and consequently reduces the training time. First, a preprocessing stage filters the raw point cloud data to a BEV map within a specific field of view. Second, the transfer learning stage uses knowledge from the previously learned classification task (with more data for training) and generalizes the semantic segmentation-based 2D object detection task. Finally, 2D detection results from the BEV image have been back-projected into 3D in the postprocessing stage. We verify results on two datasets: the KITTI 3D object detection dataset and the Ouster LiDAR-64 dataset, thus demonstrating that the proposed method is highly competitive in terms of mean average precision (mAP up to 70%) while still running at more than 30 frames per second (FPS).

## 1. Introduction

The point cloud is becoming more and more critical for autonomous driving due to the availability and significant improvement of automotive LiDAR sensors in recent years. The LiDAR sensor is capable of representing the surrounding vehicles in 3D form. It has the advantage of providing depth information and direct distance measurement, which make it suitable for autonomous driving applications such as 3D object detection [1], tracking [2], and road lane line detection [3]. However, recognizing 3D objects in LiDAR data is still challenging because LiDAR can output millions of point clouds per second, consequently increasing computational cost and effect efficiency. Alternatively, the existing methods based on point cloud address this problem using the recent deep learning techniques [4,5,6]. However, most of these methods take a supervised fashion, which means it needs large labeled data for training which can be challenging to gather and label in a specific scenario. Therefore, it is worth finding ways to improve real-time efficiency, reduce the requirements of a larger dataset, and shorten training time.

Researchers proposed the idea of semi-supervised learning [7] and weakly supervised learning [8] to solve the problem of larger annotated datasets in the 3D object detection domain. Still, these methods take annotations for a specific class of objects, and their accuracy is highly dependent on the labels in the image domain. Therefore, it is not possible to implement these methods on only LiDAR data. Existing studies on transfer learning mainly focused on 2D [9,10] object detection. Nevertheless, 2D object detection does not provide depth information, a requisite for autonomous driving tasks such as path planning [11] and collision avoidance [12].

For this reason, in this paper, a transfer learning semantic segmentation based 3D object detection approach has been proposed to take advantage of a pretrained model to minimize the need for large annotated datasets and consequently reduce training time requirements. Unlike conventional methods, the proposed architecture has been trained on a small dataset for a short period. Still, the model achieves real-time efficiency with mean average precision (mAP) up to 70%. During experiments, it is observed that a pretrained model is beneficial for a classification task and can also convey knowledge to other tasks such as the semantic segmentation task with certain modifications. Hence, the fully connected layers are changed into convolution layers, enabling a classification network to output a heat map. The proposed network architecture is based on an encoder-decoder strategy, where MobileNetv2 [13] is used as an encoder and builds a simple yet efficient lightweight matching decoder on the top. To avoid losing low-level information, skip-connections similar to U-Net [14] have been added. Finally, a modified cross-entropy loss has been defined that takes unlabeled pixels into account through masking. The main contributions of this paper are:

1. A 3D point cloud is projected into birds-eye-view representation with filtering techniques to improve the model learning capability and reduce computing time.
2. The amount of annotated data and time required for training has been minimized using transfer learning.
3. The proposed model runs in real-time, which is almost two times faster than many of the leading 3D object detection methods for LiDAR.

The famous KITTI vision benchmark suite [15] and dataset collected around the campus using Ouster LiDAR-64 has been used for 3D object detection and birds-eye-view 2D object detection.

The rest of the paper is organized as follows: Section 2 presents related work, Section 3 explains the material and proposed method, Section 4 contains results and discussion, and finally, Section 5 concludes the paper and gives the road map for future work.

## 2. Related Work

There are various methods available for object detection. Depending upon the approach used, they are divided into the following three categories:

### 2.1. 3D Object Detection

Three-dimensional object detection systems aim to detect objects of interest and estimate oriented 3D bounding boxes in the 3D real world. However, existing methods are based on full supervision, assuming that precise 3D ground truth is provided in the training dataset. Frustum-based Networks [16] using PointNet [17] deal directly with the point cloud shown high performance on KITTI benchmark suite [15] in birds-eye-view (BEV) detection category. However, the model has two downsides: (i) The model detection accuracy depends on the camera used as a secondary sensor. (ii) The framework runs two deep learning pipelines simultaneously, resulting in lower efficiency.

In contrast, VoxelNet [18] operates only on LiDAR data. The whole architecture is implemented in an end-to-end manner without any preprocessing. During training, grid cell inside features are learned using a PointNet [17] approach to build up a Convolutional Neural Network (CNN) that predicts 3D bounding boxes. Regardless of high accuracy, the model has a low inference rate of only 4 FPS on TitanX GPU [18]. McCrae [19] minimizes the number of LiDAR frames per forward pass by modifying PointPillars to become a recurrent network. Furthermore, the model accuracy has been increased to detect smaller objects more accurately.

Methods such as [20,21] propose the idea of multi-sensor fusion networks [20,21] to increase the model accuracy, but despite high accuracy, these methods are computationally expensive. To tackle the sensor-fusion computational problem, this [22] proposed an early-fusion method to fuse both camera and LiDAR with only one backbone, attaining a good balance between accuracy and efficiency. Other methods such as [23] solve the problem of data correction and temporal point cloud fusion for object detection using only 4-layer LiDAR. However, the discussed state-of-the-art approaches’ performance depends on the large-scale training dataset and ground truth labels.

### 2.2. Semi-Supervised Object Detection

Semi-supervised object detection assumes that annotation for the bounding boxes is not included in the training dataset and trained model only with image-level labels. Minsu et al. [24] apply the idea of weakly supervised learning and propose a part-based region matching approach to output a set of candidate bounding boxes for object and object parts. Sangineto et al. [25] suggest a training protocol based on a self-paced learning pattern. Bilen [26] changes the image classification network to anticipate object proposal selection and classification at the region level. Object instance mining (OIM) framework [27] detects all possible object instances in each image by initiating information propagation on all the spatial appearance graphs without additional labeling. This work [28] gives the idea of unsupervised learning for object detection utilizing weighted cluster as a separate cluster but failed to put 3D bounding volumes on a detected candidate. Recently, we have seen many state-of-the-art semi-supervised and unsupervised based object detection; however, most of the detectors are for 2D object detection rather than 3D. Extracting 3D bounding boxes without full supervision, especially from the point cloud, is still a challenging and ill-efficient approach.

### 2.3. Transfer Learning

Transfer learning uses the knowledge gained while solving one problem (where we have access to a larger dataset) and applies it to a different but related problem (where we have a limited dataset). Methods like [29] used transfer learning for semantic segmentation to minimize the gap between abundant synthetic data and limited real data.

Unlike existing weakly supervised approaches, Hong et al. [30] proposed a decoupled encoder-decoder architecture to generates spatial highlights of each class presented in images using an attention model and eventually perform binary segmentation for each highlighted region using the decoder. Lokesh [31] uses transfer learning to overcome the classification problem. The transfer learning-based approach is a well-known research topic in the 2D detection domain, whereas 3D object detection based on transfer learning technique is a much less explored and more challenging topic, especially on point cloud data. There are few works available but they will be further explored in this paper.

## 3. Proposed Approach

This section describes the point cloud grid-based preprocessing and the specific network architecture. The ultimate goal is to find an optimal policy π that maps the point clouds *p* to an obstacle information obj, which includes the position $P_{\left(x,y\right)}$ and distance d∈R. Unlike many traditional 3D object detection methods, we do not rely on a large labeled dataset. Alternatively, the proposed method is trained on a minimal dataset to map the 3D point cloud into a 2D image frame, making it faster and more reliable.

(1) obj=π(p)

**Figure 1:**
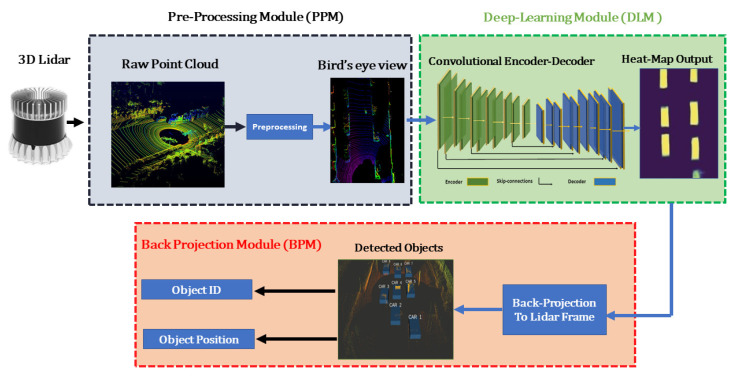
Overview of the proposed 3D object detection architecture. The proposed model directly utilizes LiDAR-based birds-eye-view (BEV) images to estimate and localize 3D bounding volumes. The whole pipeline consists of a preprocessing module, deep learning, and back projection module.

As shown in the Figure 1, the detection pipeline consists of three modules, (1) preprocessing module (PPM), (2) deep learning module (DLM), and (3) back projection module (BPM), which is further elaborated in the coming sections.

### 3.1. Preprocessing Module (PPM)

**Figure 2:**
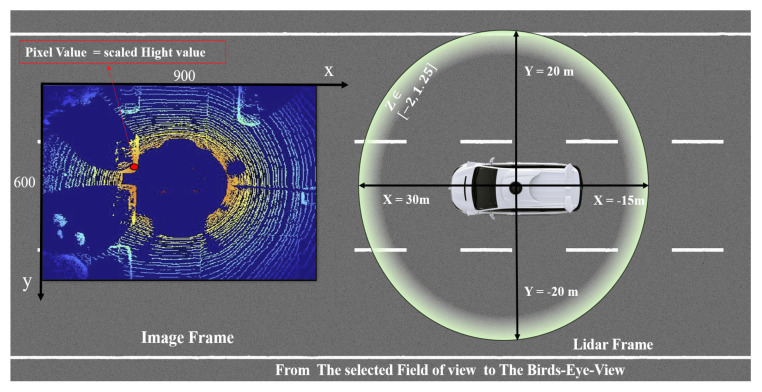
Schematic representation of the birds-eye-view RGB map.

The 3D point cloud $p=\left[\begin{array}{c} x\\ y\\ z \end{array}\right]$ inside covering area Φ of a single frame obtained from LiDAR is converted into a single birds-eye-view RGB map $b_{t}$ as shown in the Figure 2. The grid size of the image $b_{t}$ is defined with n=600 and m=900 and resolution of about γ = 10 cm.

The LiDAR is considered within the origin with respect to $\left[x,y\right]$ of $p_{\Phi }$ and defined:

(2) $$\begin{array}{c} p_{\Phi }=\left\{p={\left[x,y,z\right]}^{T}x\in \left[-15,30\right],y\in \left[-20,20\right],z\in \left[-2,1.25\right]\right\} \end{array}$$

(3) $$\begin{array}{c} x_{bt_{img}}=-p\left(y\right)*\gamma \end{array}$$

(4) $$\begin{array}{c} y_{bt_{img}}=-p\left(x\right)*\gamma \end{array}$$

(5) $$\begin{array}{c} P_{z}=[\left(\left(max_{z}-p\left(z\right)/\left(max_{z}-min_{z}\right)\right)*255\right] \end{array}$$

where in Equations (2) and (3) each pixel position was defined from the $p_{=}{\left[x,y\right]}^{T}\in p_{\Phi }$ coordinates. The pixel value $P_{z}$ in Equation (5) is the normalized *z* component of the point cloud partitioned into three regions, yielding a three-channel 2D representation.

### 3.2. Deep-Learning Module (DLM)

The Deep-Learning Module (DLM) takes as input the generated birds-eye-view image $b_{t}$ from the PPM. The DLM leverages knowledge from a pretrained classification model and uses it to solve semantic segmentation for 3D object detection. Traditional deep learning models are designed to work in isolation. These models are task-specific, which means that the model has to be trained from scratch once the domain changes.

**Figure 3:**
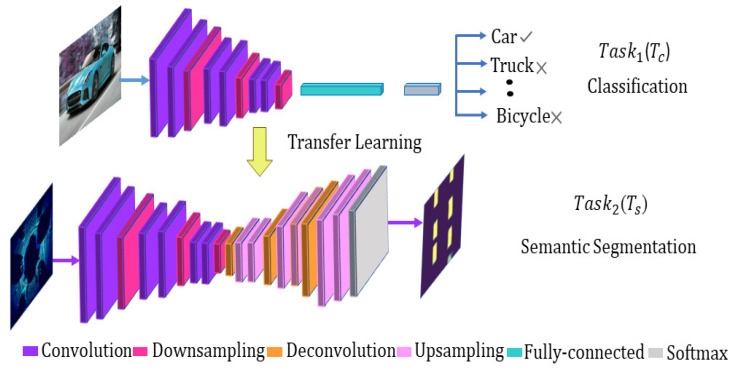
Transfer learning from classification to segmentation.

Transfer learning is the idea of overcoming the isolated learning paradigm and utilizing knowledge acquired from one task to solve related ones. Certain low-level features can be used for a task different from the original one in computer vision applications. For transfer learning implementation, first a classification function $F_{c}$ has been defined as:

(6) $$F_{c}=\left\{\left(c_{1},y_{1}\right)\ldots \left(c_{n},y_{n}\right)\right\}$$

where, $c_{i}$ ∈ *C* is the number of samples for training and $y_{i}$ ∈ *Y* are the corresponding labels for each class, a predictive function $f_{q}$, which can be presented probabilistically as p(y/c), and output a classification task $T_{c}$. Second, we define the semantic segmentation function $F_{s}$ as:

(7) $$F_{s}=\left\{\left(s_{1},l_{1}\right)\ldots \left(s_{n},l_{n}\right)\right\}$$

where, $s_{i}$ ∈ *S* is the number of samples used for training the segmentation model and $l_{i}$ ∈ *L* are the labels for each class and finally a segmentation predictive function $f_{k}$ and specific segmentation task $T_{s}$. Using transfer learning, we improve the learning of segmentation predictive function $f_{k}$ using knowledge in $F_{c}$ and $f_{q}$ where, $T_{c}$ ≠ $T_{s}$ as shown in Figure 3. Knowledge from the pretrained classification task act as an additional input when learning a semantic segmentation task.

Equations (6) and (7) defined for transfer learning are used to convert the classification network into a convolutional network to output a segmented heat map. The segmented heat map later provides the basis for the birds-eye-view 2D object detection. The CNN models trained for image classification restrain relevant information used for segmentation in the proposed method. The convolution layers of the pretrained model are reused in the encoder layer of the segmentation model. Another primary requirement for transfer learning is the availability of pretrained models. Luckily, the deep learning community open-sourced many of the pretrained models such as VGG-16 [32], Inception [33], Deeplab [34], and MobileNet. The MobileNetv2 pretrained model has been adopted in the proposed method because of its significant fever parameters and minor computational complexity.

The model architecture usually consists of several convolutional layers, nonlinear activations, batch normalization, and pooling layers. The initial layers tend to learn the low-level concepts and the higher layers retain the high-level information. For the image classification task, the model maps the spatial tensor from the convolution layer to a fixed-length vector using fully connected layers which flatten all the spatial information.

In contrast, for semantic segmentation, spatial information is critical. Therefore, the fully connected layers are converted into convolutional layers. The DLM is based on encoder-decoder architecture where at the encoder stage, the convolutional layers combined with downsampling layers produce a low-resolution tensor containing the high-level information, and at the decoder stage, more convolutional layers have been added and coupled with upsampling layers to increase the size of the spatial tensor and generate high-resolution segmentation outputs.

**Figure 4:**
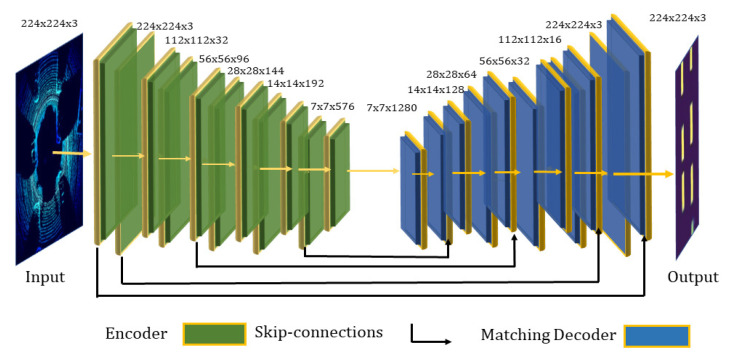
Schematic illustration of an encoder-decoder architecture. The left-hand side is a birds-eye-view RGB map that is passed to a series of computational layers, and the right-hand side is the output decoder feature map. The arrows are skip connection layers, where input is being directly concatenated from encoder to decoder.

However, simply stacking the encoder and decoder may result in the loss of low-level information. Hence, the segmentation map boundaries generated by the decoder will be defective. Therefore, the decoder has been allowed to access the low-level features produced by the encoder layers using skip connections. Intermediate outputs of the encoder are concatenated with the inputs to the intermediate layers of the decoder at relevant positions, as shown in Figure 4.

Table 1 presents the general framework of the MobileNetV2, where the number of the output channel is represented by *c*, *n* means repeating number, *s* is the stride, and for the spatial convolution, 3×3 kernel has been used. The network of width 1,224,224 uses 3.4 million parameters with a resulting computational cost of 300 million multiply-adds. Even the architecture size ranges between 1.7 million and 6.9 million, and the computational cost reaches less than 585 million MAdds.

### 3.3. Back Projection Module (BPM)

After generating the heat map *h* from the DLM, postprocessing is required to extract 2D rectangles from the generated heat map and lift those 2D rectangles into 3D bounding volumes in the LiDAR frame. In Equation (8) Canny edge detector [35] has been used for thresholding to translate the generated heat map into 2D rectangles. The output image $h_{\left(x,y\right)}$ was obtained from the original image ${\mathrm{bt}}_{\left(x,y\right)}$ as:

(8) $$\left\{\begin{array}{c} 1\;if\;{\mathrm{bt}}_{\left(x,y\right)}⩽Th\\ 0\;if\;{\mathrm{bt}}_{\left(x,y\right)}>Th \end{array}\right.$$

where (x,y) is the coordinate of threshold Th.

Then contour has been used for the respective binary masks and the minimal bounding rectangles of those contours. These 2D bounding boxes provide us the basis for 3D bounding volumes. After getting the 2D bounding rectangles, five of the seven parameters that define the 3D bounding box have been extracted. The estimated parameters are the $ob_{x}$, $ob_{y}$, $P_{z}$ location, and rotation on the image plane.

The proposed framework estimates the height information directly without regression to convert the 2D rectangle into 3D bounding volumes. The model runs with a fixed height location extracted from the ground truth. It is assumed that objects are on the ground, and this assumption is reasonable in the autonomous driving scenario as vehicles do not fly [36].

The back projection module (BPM) is used to project 2D information from the heat map coordinate $h_{xyz}$ into the LiDAR coordinate $l_{xyz}$ as follows:

(9) $$\begin{array}{c} l_{x}=-ob_{x}/\gamma \end{array}$$

(10) $$\begin{array}{c} l_{y}=-ob_{y}/\gamma \end{array}$$

(11) $$\begin{array}{c} l_{z}=P_{z}*\left(max_{z}-min_{z}/255\right)+min_{z}+1.8 \end{array}$$

where $ob_{x}$, $ob_{y}$ are the detected position of the objects in $h_{xy}$ heat map frame, and $P_{z}$ is the normalized pixel value of image $b_{t}$. $l_{x}$, $l_{y}$, and $l_{z}$ are the estimated object position in the LiDAR frame. A constant value of 1.8 has been added in Equation (9) considering the LiDAR position on the top of the car. Finally, to estimate the distance from the detected object, the Euclidean distance formulae has been used as:

(12) $$d=\sqrt{l_{x}^{2}+l_{y}^{2}}$$

A complete workflow for the presented approach is shown below in Algorithm 1.
**Algorithm 1** Transfer learning based semantic segmentation for 3D object detection.preprocessing module (PPM):- Specify the field of view FOV in x,y,z axis- Apply the filter to FOV- Generate the birds-eye-view RGB map $b_{t}$deep learning module (DLM):- Apply the transfer learning based semantic segmentation to the $b_{t}$ and get the heat map *h*- Apply the Canny edge detector on the heat map *h*- Detect the all counters *C* as 2D rectanglesfor *C* = 1,2,3 *…*, M - Get the pixel position $\left(ob_{x},ob_{y}\right)$ of each objectback projection module (BPM):- Project the $ob_{x},ob_{y}$ to the LiDAR frame $\left(l_{x},l_{y},l_{z}\right)$ by following the equations 9→11- Calculate the distance *d* from each object

## 4. Experiments

This section assesses the performance of the proposed algorithm. The experiment is carried out as follows: First, we describe the proposed architecture experimental setup. Second, segmentation results are compared before and after implementing transfer learning, and finally, a m odel efficiency comparison is made against mean average precision (mAP) with conventional methods in the LiDAR frame.

### 4.1. Experiment Setup

#### 4.1.1. Real-Time Validation Using the Ouster LiDAR-64

The 3D laser scanner used in this experiment is Ouster LiDAR-64. The operating system used for data collection, training, and evaluation is Ubuntu-16. The robot operating system (ROS) Kinetic environment has been used for experimenting. The central processing unit i5-6500 and the graphic processing unit 1050ti are used for training. For data collection and evaluation, a dedicated Intel mini-pc (NUC-10i7) has been used. The data for the experiment was gathered around the campus of Kunsan National University.

#### 4.1.2. Real-Time Validation Using KITTI Dataset

For training and evaluating the proposed architecture, another dataset used is the famous KITTI object detection dataset. The public KITTI dataset provides 7481 point cloud samples for training and 7518 for evaluation using Velodyne-64. Unlike existing methods for 3D object detection, the proposed model used only 824 samples for training and 256 frames for validation, as shown in Table 2. The model has been trained for 40 epochs with a learning rate of 0.0003. The learning rate is kept smaller to avoid losing previously learned knowledge, and it also will ensure that CNN weights do not distort too early.

### 4.2. Experimental Results

#### 4.2.1. Transfer Learning vs. Scratch Training

**Figure 5:**
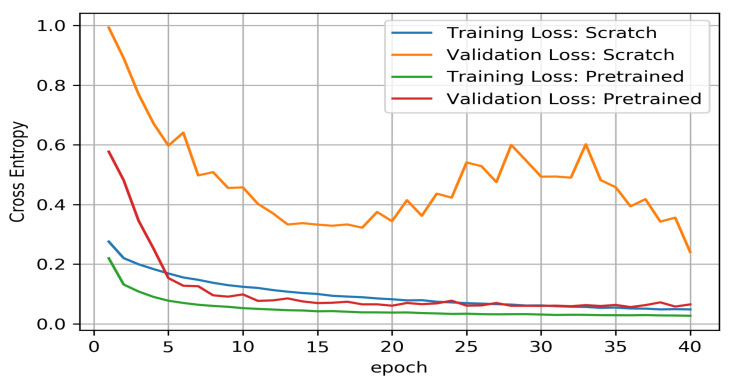
Comparison between model from scratch and model initialized with pretrained classification weights.

The proposed model is trained first from scratch, without using any pretrained weights. However, it is hard to train a significant architecture such as MobileNet-v2 and accompanying decoder robustly on a minimal dataset. As a result, training from scratch leads to very strong overfitting, as shown in Figure 5. The architecture is highly parameterized, and it is challenging for a model to learn with a few examples without prior knowledge.

**Figure 6:**
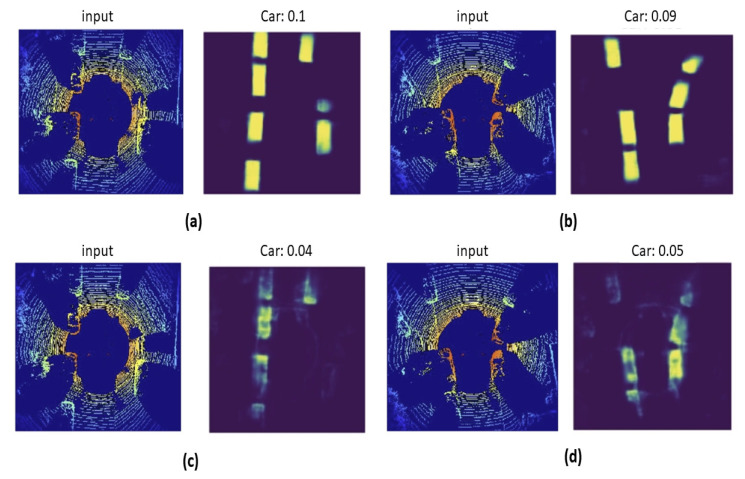
Comparison between model from scratch and model initialized with classification pretrained weights: (**a**,**b**) shows prediction using pretrained weights, (**c**,**d**) shows prediction using the model trained from scratch.

In the second experiment, the same architecture is used but initialized with ImageNet classification pretraining weights. As shown in Figure 5, the initial losses are considerably lower and converge faster both in training and validation. The predicted heat maps in both scenarios are shown in Figure 6, Figure 6a,b show the prediction result when the model is initialized with pretrained weights, and Figure 6c,d show the prediction result when the model is trained from scratch. Results verify that the model initialized with pretrained weights shows higher accuracy than the model trained from scratch.

#### 4.2.2. Trained Model Evaluation

**Figure 7:**
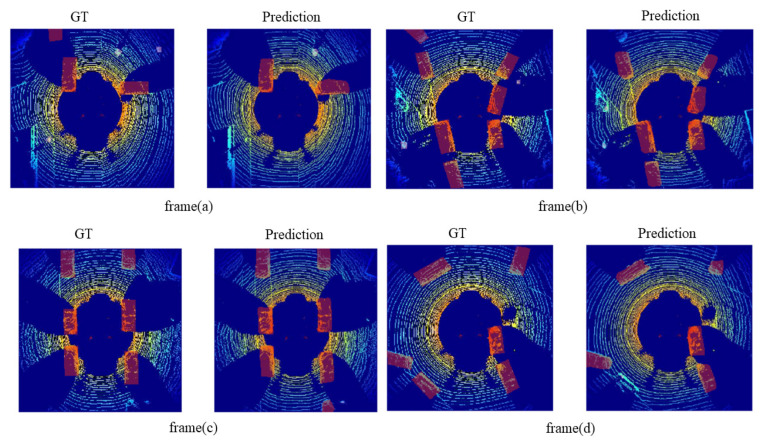
The visualization results on KITTI dataset using our proposed method. Subfigures (**a**–**d**) shows the ground truth on the right-hand side, and the left-hand side shows the output results. The images shows that the proposed model performs well in different scenarios.

The Intersection over Union (IoU) metric, also known as the Jaccard index, is essentially a method to quantify the percent overlap between the target mask and the prediction output. The metric range is defined between 0% and 100% with 0% indicating no overlapping and 100% representing a perfect overlapping. Figure 7 shows the segmented ground truth and the predicted output. Table 3 contains the IoU for the frames shown in the Figure 7, where the model achieves an average IoU around 90%.

In 3D point clouds, the sparsity increases as the distance between the LiDAR and detected object increases. Therefore, the amount of points reduces for an object of a similar class and the same size. The proposed architecture has been tested against different distances. We designed different filters to cover different ranges and analyze their impact on model performance. It is observed that the model performed well while covering objects within ranges from −15 to 35 m in the longitudinal direction and −20 to 20 m in the lateral direction around the vehicle, which is followed by experimental testing.

#### 4.2.3. Object Detection Results

**Figure 8:**
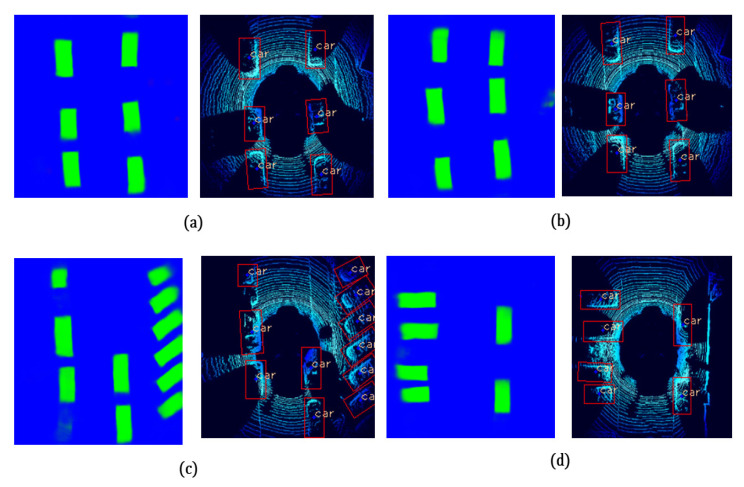
(**a**–**d**) shows samples of prediction on the left-hand side and extracted contours for the car class on the right-hand side. Subfigures (**a**,**b**) shows the simple scenarios, and (**c**,**d**) shows the images where the proposed model is able to achieve accurate results in more complex scenarios with rotated bounding boxes.

The proposed method has been validated using the KITTI dataset. Figure 8 shows the obtained heat map results from the segmentation model. Thresholding and contours techniques are used to extract the 2D bounding box coordinates in the birds-eye-view image frame.

**Figure 9:**
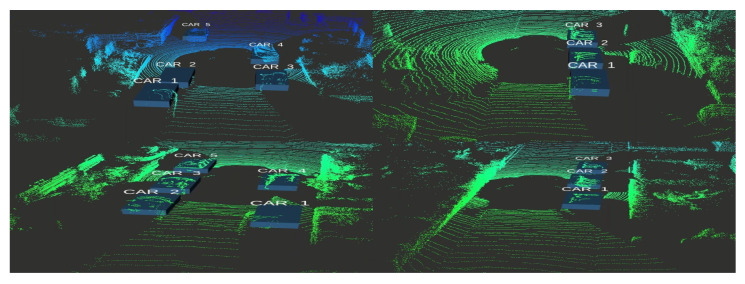
Qualitative results of the proposed model using KITTI 3D object detection dataset in LiDAR frame.

**Figure 10:**
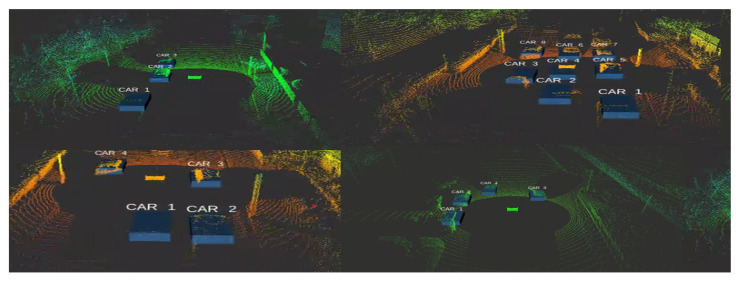
Qualitative results of the proposed model using the Ouster LiDAR-64 dataset in LiDAR frame.

Moreover, the obtained 2D coordinates have been used in the back projection module (BPM) to extract the 3D obstacle information and display it as a 3D bounding volume using Equations (9)–(11). Qualitative results of the proposed model are shown in the figures below where Figure 9 shows 3D object detection results from the KITTI dataset and Figure 10 shows 3D object detection results from the Ouster Lidar-64 dataset.

**Figure 11:**
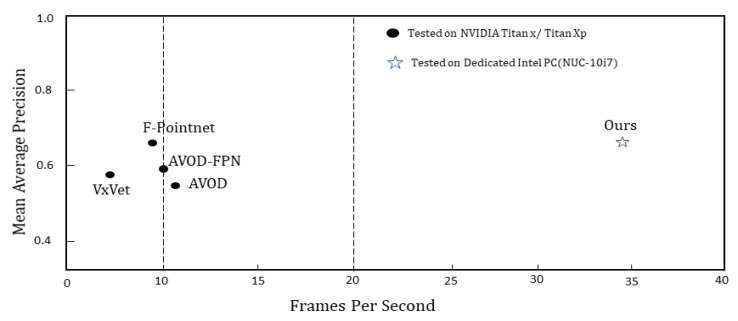
Performance comparison. This plot shows the mean average precision (mAP) against the run-time (FPS) in the LiDAR frame. We compare our proposed model with the existing model for 3D object detection and measured our architecture performance on a dedicated embedded platform (Intel PC) with real-time efficiency.

We compare our model with existing approaches for 3D object detection in terms of efficiency, which are essential for autonomous vehicles and where most state-of-the-art algorithms struggle. Figure 11 presents the comparison results. The proposed framework runs at more than 30 frames per second (FPS) on a dedicated platform without a GPU system.

## 5. Conclusions

In this paper, we present a real-time 3D object detection that utilizes LiDAR point cloud. The proposed method takes birds-eye-view representation as input for computation efficiency. The overall pipeline is defined as an end-to-end manner in which the preprocessing modules (PPM) take the LiDAR data and convert it into a birds-eye-view (BEV) image. The deep learning module (DLM) takes the BEV and outputs a segmented heat map that transforms into 3D bounding volumes in the postprocessing module (PPM). We highlight the proposed approach results in terms of accuracy (Figure 11) with excellent efficiency of more than 30 frames per second (FPS). We do not need a large and well-labeled dataset for training, similar to most of the leading approaches. This breakthrough is achieved by the introduction of the deep learning technique called transfer learning. Experiments demonstrate that the proposed method can potentially reduce the need for a large, well-labeled dataset and facilitate the implementation of 3D object detectors in new scenarios. Future work is planned to develop a model that takes raw point cloud as input and uses the sensor fusion to achieve breakthroughs in both accuracy and speed.

## Figures and Tables

**Table 1:** MobileNetV2 overall architecture [13].

Input	Operator	*t*	*c*	*n*	*s*
$224^{2}\times 3$	conv2d	-	32	1	2
$112^{2}\times 32$	bottleneck	1	16	1	1
$112^{2}\times 16$	bottleneck	6	24	2	2
$56^{2}\times 24$	bottleneck	6	32	3	2
$28^{2}\times 32$	bottleneck	6	64	4	2
$14^{2}\times 64$	bottleneck	6	96	3	1
$14^{2}\times 96$	bottleneck	6	160	3	2
$7^{2}\times 160$	bottleneck	6	320	1	1
$7^{2}\times 320$	conv2d 1 ×1	-	1280	1	1
$7^{2}\times 1280$	avgpool 7 ×7	-	-	1	-
1×1×1280	conv2d 1 ×1	-	K	-	

**Table 2:** Comparison between the existing methods and proposed method in terms of data requirements and training time.

Model	Modality	FPS	No. Frmaes (Training)	Avg. Training Iterations
F-PointNET [16]	LiDAR + RGB	5.9	7518	200
VoxelNet [18]	LiDAR	4.3	7518	150
FA3OD [22]	LiDAR + RGB	17.8	7518	80
MV3D [37]	LiDAR + RGB	2.8	7518	20 K
AVOD [38]	LiDAR + RGB	12.5	7518	30 K
Ours	LiDAR	30.6	824	40

**Table 3:** The IoU metric measures the overlap percentage between the ground truth and prediction.

No. Frames	IOU
frame (a)	0.921
frame (b)	0.953
frame (c)	0.972
frame (d)	0.876

## Data Availability

Not applicable.
